# Influence of the Chemical Structure on the Mechanical Relaxation of Dendrimers

**DOI:** 10.3390/polym15040833

**Published:** 2023-02-08

**Authors:** Nadezhda N. Sheveleva, Andrei V. Komolkin, Denis A. Markelov

**Affiliations:** St. Petersburg State University, 7/9 Universitetskaya nab., 199034 St. Petersburg, Russia

**Keywords:** dendrimer, shear-stress relaxation, molecular dynamics simulation, PAMAM, PPI

## Abstract

The rheological properties of macromolecules represent one of the fundamental features of polymer systems which expand the possibilities of using and developing new materials based on them. In this work, we studied the shear-stress relaxation of the second generation PAMAM and PPI dendrimer melts by atomistic molecular dynamics simulation. The time dependences of relaxation modulus *G*(*t*) and the frequency dependences of the storage *G′(ω)* and loss *G″(ω)* moduli were obtained. The results were compared with the similar dependences for the polycarbosilane (PCS) dendrimer of the same generation. The chemical structure of the dendrimer segments has been found to strongly influence their mechanical relaxation. In particular, it has been shown that hydrogen bonding in PAMAM dendrimers leads to an entanglement of macromolecules and the region is observed where *G′(ω)* > *G″(ω).* This slows down the mechanical relaxation and rotational diffusion of macromolecules. We believe that our comprehensive research contributes to the systematization of knowledge about the rheological properties of dendrimers.

## 1. Introduction

Dendrimers are hyperbranched synthetic macromolecules with a perfectly symmetrical tree-like structure [1]. Dendrimers were first synthesized in the 70s [2], but recently they have attracted more attention from scientists [3,4]. The reason for this is the global demand for new nanomolecules and materials based on them, which can be used in advanced technologies [5,6] and medicine [7]. Therefore, the optimization of synthesis methods and the development of new strategies, that allow the creation of dendrimers with different morphology and functionalization, contribute to the emergence of new types of dendrimers [8,9,10]. The behavior of dendrimers in solution remains the most studied due to their use in biomedical applications [11,12,13] as nanocontainers for gene and drug delivery [14,15,16]. New developments are underway to create novel materials based on dendrimer macromolecules and to use dendrimers as nanomodifiers for already utilized materials [17,18].

The development of the theory of mechanical relaxation of dendrimers began with analytical studies using a simplified viscoelastic model [19,20,21,22,23,24,25,26,27,28,29]. However, within the framework of this model, it is quite difficult to predict the effects determined by volume interactions, the mobility of the dendrimer as a whole, and the intermacromolecular mobility/interactions, as well as the influence of the dendrimer chemical structure. Thus, in the first experimental rheological studies of dendrimers or hyperbranched polymers, the analysis of experimental data was usually carried out without taking into account the specific tree-like topology of macromolecules [30,31,32]. In the last decade, the experimental [33,34,35,36,37,38] and molecular dynamics simulation [39,40,41,42,43,44,45] studies have shown that melts of dendrimers of various architectures have unique properties (including rheological ones).

Among the huge variety of dendrimers, polycarbosilane dendrimers, due to the absence of specific interactions, e.g., ionic and hydrogen bonds, proved to be suitable for studying the role of dendrimer architecture on physical and dynamic properties and are used as model objects. In particular, the dendrimer architecture is reflected in the relaxation times, which can be associated with inner relaxation, as well as with the relaxation of branches as whole, following the qualitative assumptions of the theory [43,46,47,48,49]. Taking into account that the total relaxation of the dendrimer branches depends on their molecular weight, the spectrum of the corresponding relaxation times is wide.

Atomistic simulation of carbosilane dendrimer melts has been performed quite recently [41,43,50]. Other types of dendrimer melts were simulated in [42,44], and coarse-grained nonequilibrium molecular dynamics simulation was used to study the shear viscosity [51,52] and the rheological properties of dendrimer melts [53].

In the recent works, molecular dynamics simulations of functionalized [54] and non-functionalized [40] carbosilane dendrimers in melts have been carried out to study their mechanical properties. It has been shown [54], the fourth generation functionalized carbosilane dendrimers (FD) have a region where *G′ > G″.* As a rule, this effect is attributed to the presence of entanglements between polymers. However, the latter seems unlikely in the case of carbosilane dendrimers, since there are no specific interactions, and the size and molecular weight are insufficient for the physical entanglements. An analysis of the structural and equilibrium properties of FD suggests that the observed effect is due to the fact that dendrimer macromolecules are in a crowded environment caused by internal densification. It has been established that dynamic characteristics indicate the following manifestations of the crowded environment in FD G4: (i) the rotational diffusion of the dendrimer does not slow down and corresponds to the size of the dendrimer (i.e., the ratio of the rotation time of the dendrimer as a whole to the cube of the radius of gyration, *τ_rot_/R_g_^3^*, has similar values for FD G3 and G4; (ii) the terminal (maximal) mechanical relaxation time, *τ_max_*, increases significantly, e.g., the ratio *τ_max_/τ_rot_* increases more than two times compared to FD G3.

On the other hand, in melts of hyperbranched polyglycerols (hbPG) with a high molecular weight, the entanglement effect was experimentally observed [55]. The authors of this Ref. [55] concluded that such macromolecules can form entanglements at a molecular weight, *M*, > 10,000 g/mol, which is significantly lower than the minimum *M* values required for linear polymer chains. It has been suggested that the main mechanism of entanglements is related to the formation of hydrogen bonds between macromolecules.

**Figure 1:**
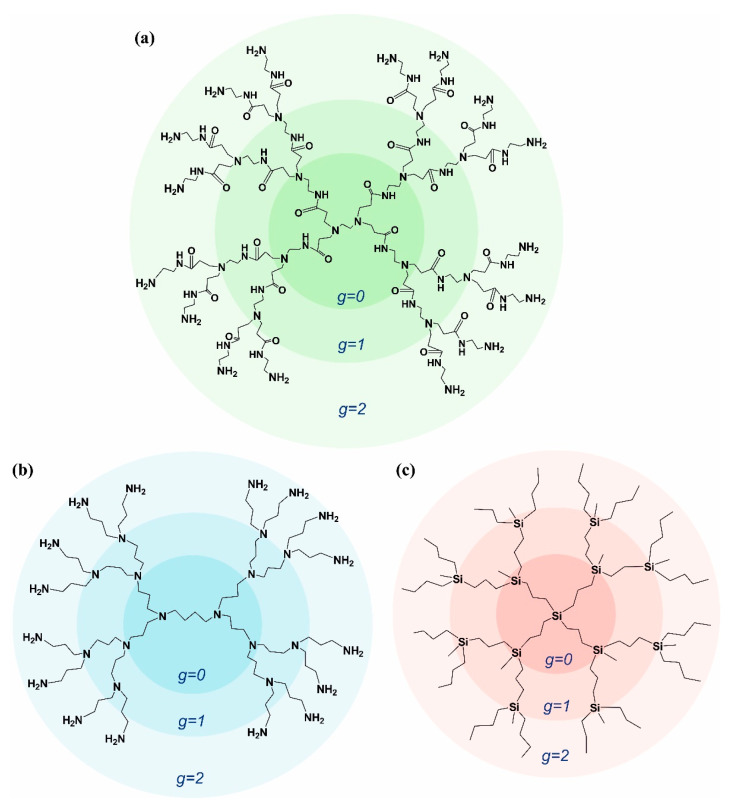
Structures of the second generation dendrimers (G = 2, with 16 terminal groups) (**a**) polyamidoamine (PAMAM), (**b**) polypropylene imine (PPI) and (**c**) polycarbosilane (PCS) dendrimers. *g* is the generation layer number.

The aim of this work is a comprehensive study of the mechanical properties of dendrimers with different chemical structures, which have not yet been carried out. We consider the influence of the chemical structure on the mechanical relaxation of dendrimers. For this purpose, we have performed atomistic molecular dynamics (MD) simulations of polyamidoamine (PAMAM) and polypropylene imine (PPI) dendrimers of the second generation (G2) in a melt (see dendrimer structures in Figure 1). For comparative analysis, we use the MD simulation results for the polycarbosilane (PCS) dendrimer obtained by us earlier in [40].

## 2. Simulation Details and Theoretical Approach

In this work, we studied PAMAM and PPI dendrimers of the second generation (G2, with 16 terminal groups) in a melt using MD simulation. The atomistic model of united atoms was used, in which only ionized hydrogen atoms in NH_2_ groups were explicitly taken into account. The simulation box with periodic boundaries contained 27 dendrimers for each system. Molecular dynamics simulations were conducted with the GROMACS package [56] and the OPLS force field [57].

At the preliminary stage of equilibration, the systems were maintained at a constant temperature of 600 K with the V-rescale thermostat [58], which was triggered every 0.1 ps, and at a constant pressure of 1 atm by using the Berendsen barostat [59] with *τ*_p_ = 1 ps during 100 ns for PPI and 500 ns for PAMAM. The lengths of these trajectories were enough for the systems to “forget” the initial configurations. Then, each system was simulated in the NPT ensemble using the Langevin thermostat at different values of the coupling constant *τ_T_* = 0.005, 0.05, and 0.5 ps in order to vary the friction in the system. For the highest value of *τ_T_*, we simulated ten replicas in order to have better statistics at long times. Each system was equilibrated at least for 100 ns. The final trajectories were obtained in NVT ensemble during 600 ns for PPI and 1200 ns for PAMAM dendrimers.

To calculate the mechanical relaxation, we have used the method developed in Refs. [40,54]. The dynamical modulus *G*(*t*) is calculated from the fluctuations of the shear-stress tensor $\hat{P}=\left(P_{\alpha \beta }\right)$ [60,61]

(1) $$G\left(t\right)=\frac{V}{30k_{B}T}\overset{}{\underset{\left(\alpha \beta \right)}{\sum }}\left(6\langle P_{\alpha \beta }\left(t\right)\rangle \langle P_{\alpha \beta }\left(0\right)\rangle +\langle N_{\alpha \beta }\left(t\right)\rangle \langle N_{\alpha \beta }\left(0\right)\rangle \right)$$

where *V* is the box volume, *T* is the temperature, and *k_B_* is the Boltzmann constant, *N_αβ_* = *P_αα_* − *P_ββ_*, (*αβ*) sums over the *xy*, *yz*, and *zx* components of $\hat{P}$.

The resulting *G*(*t*) obtained at different values of the parameter *τ_T_* are superimposed based on the rotational relaxation autocorrelation function,

(2) $$P_{1}^{rot}\left(t\right)=\langle \vec{u}\left(t\right)\times \vec{u}\left(0\right)\rangle$$

where $\vec{u}\left(t\right)$ is a unit vector connecting two nitrogen atoms, one nitrogen from the periphery and the other from the core. The exponential tail of the function *P*_1_^rot^(*t*) is characterized through the time *τ_rot_*. The *P*_1_^rot^(*t*) related to different *τ_T_* can be perfectly rescaled based on *τ_rot_*. The same happens for *G*(*t*).

## 3. Results and Discussions

### 3.1. Relaxation Modulus

**Figure 2:**
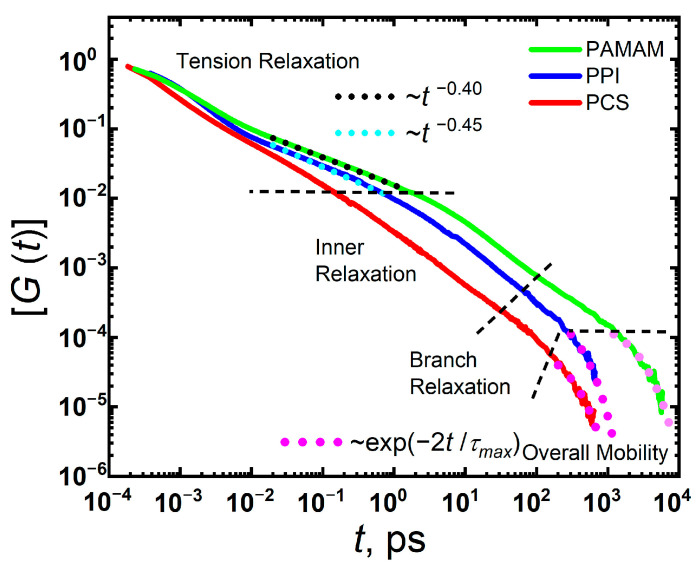
Double-logarithmic representation of the normalized shear-stress relaxation moduli [*G(t)*] = *G(t)/G(0)* for PAMAM and PPI dendrimer melts. For comparison the data for PCS melt are adapted with permission from Ref. [40]. 2019, American Chemical Society.

**Figure 3:**
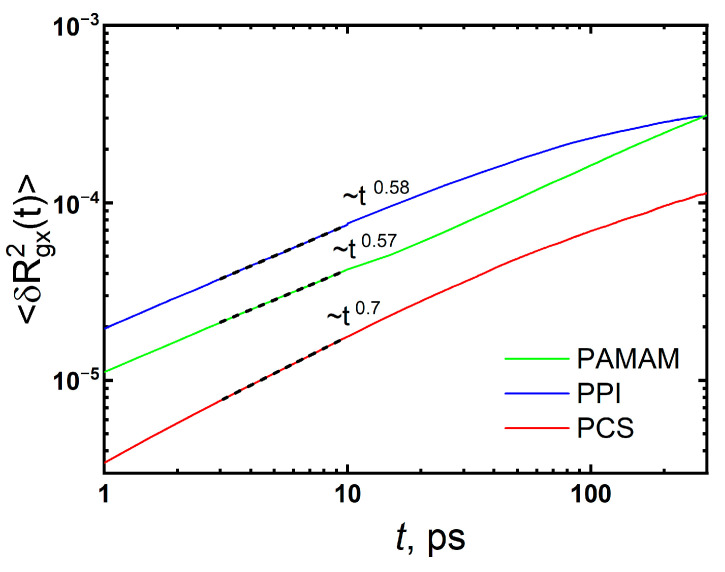
Fluctuations of the x-component of the gyration tensor ⟨δR_g,x_^2^(t)⟩ ≡ ⟨[R_g,x_(t) − R_g,x_(0)]^2^⟩ for the dendrimers. For comparison the data for PCS melt are adapted with permission from Ref. [40]. 2019, American Chemical Society.

Figure 2 shows the time dependences of the relaxation moduli, *G(t)*, for PAMAM G2 and PPI G2 dendrimers). For comparison, the *G(t)* dependence for PCS G2 is presented from our previous work (see [40]). As can be seen from this figure, the moduli *G(t)* of the studied dendrimers differ greatly from that one of PCS G2. In the region of tension relaxation, the slope of the *G(t)* dependences for PPI and PAMAM dendrimers is similar (−0.45 and −0.40, correspondingly), in contrast to −0.7 for PCS. In the case of PCS, the slope is determined by fluctuations in the x-component of the gyration tensor ⟨δR_g,x_^2^(*t*)⟩ ≡ ⟨[R_g,x_(*t*) − R_g,x_(0)]^2^⟩. The ⟨δR_g,x_^2^(*t*)⟩ functions for PPI and PAMAM are plotted on a double-logarithmic scale in Figure 3. A decrease in the slope of ⟨δR_g,x_^2^(*t*)⟩ can be observed for PPI and PAMAM to 0.57–0.58. Despite the different sizes of PPI and PAMAM dendrimers and the structures of their segments between branching points, the slopes of the *G(t)* dependences in the tension relaxation region and the slopes of ⟨δR_g,x_^2^(*t*)⟩ are almost the same. Apparently, this is due to the global conformations of PPI and PAMAM G2 in the melt, which differ significantly from PCS G2.

**Figure 4:**
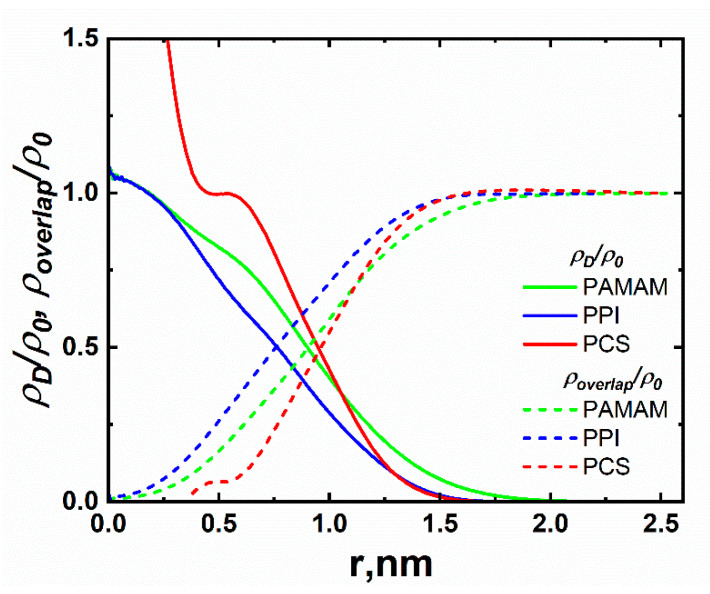
The radial density profiles from the center of mass of the macromolecule both for selected dendrimer, *ρ_D_*, (solid) and for the rest of the dendrimers in the system, *ρ_overlap_*, (dashed) using the formula: ρ(r)=<m(r)>/V(r), where *ρ*(*r*) is the average density in the spherical layer at a distance *r* from the dendrimer’s center of mass, <*m*(*r*)> is the average total mass of atoms in the layer of volume *V*(*r*).

The radial density profiles (Figure 4) show that the PCS dendrimer has a significantly denser core than the PPI and PAMAM dendrimers. Accordingly, the chemical structure (Figure 1), the core of the PCS dendrimer, has a functionality of *F_c_* = 4 (i.e., four segments are attached to the central silicon atom). As for PPI and PAMAM, the dendrimer core consists of two branching points (two nitrogen atoms) and formally has the functionality *F_c_* = 2. Therefore, due to the less dense cores, the mutual penetration of PPI and PAMAM dendrimers into the center is significantly higher than in the case of PCS dendrimers. This apparently slows down the relaxation of *G(t)* at short times. It is important to note that with increasing generations, the difference in *G(t)* at short times between PCS and the studied dendrimers may considerably decrease or disappear, since the neighboring macromolecules of the PPI and PAMAM dendrimers will not be able to penetrate into the core region.

However, the slopes of the *G(t)* curves change more strongly than those of ⟨δR_g,x_^2^(*t*)⟩. We suggest that such a change is associated not only with fluctuations in the size of the dendrimer, but also with the formation of entanglements between macromolecules due to hydrogen bonding. In addition, according to the theory based on the viscoelastic model [23], an increase in the number of entanglements between dendrimers should lead to a slowdown in relaxation at short times. We will consider this issue in detail when analyzing the frequency dependences of the storage and loss moduli.

At an intermediate region, the inner relaxation spectrum is the main contributor to the *G(t)* dependence [40]. According to the theory, the inner spectrum is rather narrow and limited by times from 0.17*τ*_0_ to 5.8*τ*_0_, where *τ*_0_ is the relaxation time of an individual terminal segment. The segment length of the PAMAM dendrimer is significantly longer than that of the PPI dendrimer. Obviously, an increase in the segment length should lead to an increase in *τ*_0_ of the segment. In the case of PAMAM, this in turn leads to a shift of the inner relaxation region towards longer times and a slower decay of the *G(t)* curve compared to PPI and PCS.

Further along the time scale, the *G(t)* dependence is determined by branch relaxation. At low generations, the branch relaxation spectrum in PCS manifests weakly compared to G > 2 (see Ref. [40]), since its characteristic times correspond to the motions of branches and/or sub-branches as a whole.

According to the theory [23], in the case of G = 2, there are only two non-degenerate times except the characteristic relaxation time of the terminal segment (i.e., *τ*_0_). This leads to a rather narrow region of branch relaxation. A similar situation is observed for PPI. However, for PAMAM, the branch relaxation region is broadened. This can be explained by the presence of entanglements within the PAMAM dendrimer due to hydrogen bonding between groups of neighboring branches/sub-branches (see Table 1). In the PAMAM dendrimer, the average number of intramolecular hydrogen bonds is 7.5, while they are absent in PPI and PCS.

At longer times, mechanical relaxation is determined by the mobility of the dendrimer as a whole and/or interactions between the dendrimer macromolecules. To analyze the relaxation of *G(t)* in this region, we used the single-exponential fitting (i.e., ~exp(−2t/*τ_max_*), where *τ_max_* is the terminal (maximal) relaxation time of the *G(t)* dependence). The *τ_max_* values are shown in Table 1 for all the dendrimers under consideration. It can be seen from this table that the time *τ_max_* for PAMAM G2 is almost an order of magnitude longer than for the other dendrimers.

In order to compare the rotational diffusion and the maximal relaxation time we consider the ratio *τ_max_*/*τ_rot_*. For all the dendrimers, the ratios *τ_max_*/*τ_rot_* have similar values. Therefore, for the studied dendrimers, the mechanical relaxation of the macromolecule as a whole corresponds to its rotational mobility. It is important to note that for PCS at G > 2, this ratio increases, which indicates a slowdown in mechanical relaxation compared to rotational diffusion of dendrimers caused by the crowded environment [54]. Thus, the difference in *τ_max_* for PAMAM G2 and for other considered dendrimers is not related to this effect.

We also calculated the ratio *τ_rot_*/*R_g_^3^* in order to take into account the difference in the sizes of dendrimers (i.e., in *R_g_*) [54,62]. However, when using the equilibrium parameter (*R_g_*^3^) to calibrate *τ_rot_*, the parameter *B* increases slightly for PPI and more than 5 times for PAMAM compared to PCS. We believe that this is caused by entanglements between dendrimers due to the formation of intermolecular hydrogen bonds, of which there are on average two in PPI and 11 in PAMAM dendrimer melts. This slows down both the maximal mechanical relaxation time and the rotational diffusion of the macromolecule.

### 3.2. Storage and Loss Moduli

**Figure 5:**
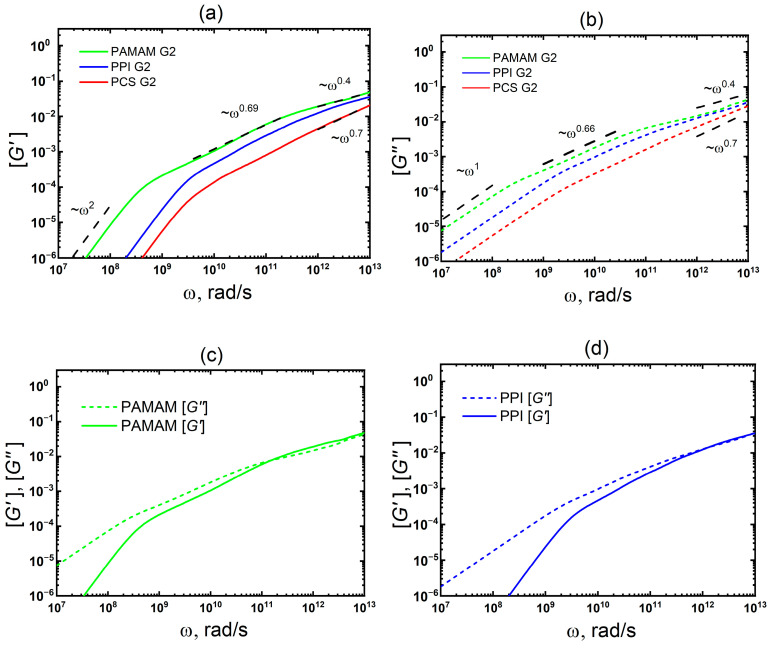
Double-logarithmic representation of the storage [G′(ω)] (**a**) and loss [G″(ω)] (**b**) moduli for PAMAM and PPI melts are plotted together; (**c**,**d**) separately for PAMAM and PPI, respectively. For comparison the data for PCS melt are adapted with permission from Ref. [40]. 2019, American Chemical Society.

Figure 5 shows the frequency dependences of the storage *G′(ω)* and loss *G″(ω)* moduli for the dendrimer melts under consideration. The growth of *G′(ω)* and *G″(ω)* in the low-frequency region is determined by *τ_max_ω^2^* and *τ_max_ω*, correspondingly. Therefore, the fastest growth in this region is observed for PAMAM. The *G′(ω)* and *G″(ω)* curves for PPI and PCS differ slightly.

In the mid-frequency range, all *G*″*(ω*) and *G*′*(ω)* curves do not intersect and have slopes of 0.66 and 0.69, respectively. These values are quite similar to the slope of 0.7 obtained experimentally for PPI dendrimers G = 3–5 (G = 2–4 in our numbering) [34]. For a more detailed comparison with experiment, MD simulation of higher generation dendrimers is required, which will be carried out in the near future.

**Figure 6:**
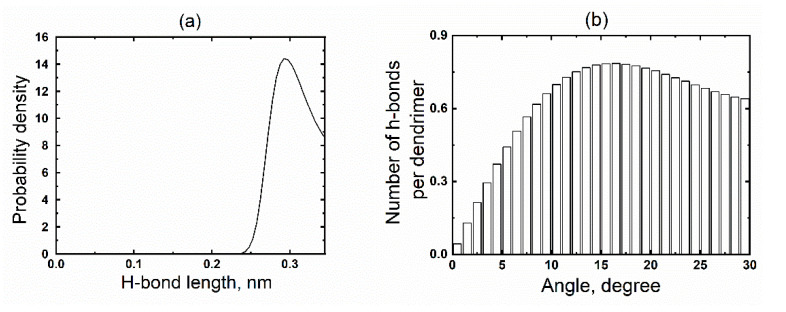
The hydrogen bond length (**a**) and angle (**b**) distributions in PAMAM G2 dendrimers in the melt.

It is important to note that in the case of FD G4, in this frequency range (10^6^–10^8^ rad/s), a region was observed where *G′(ω)* > *G″(ω)*. Moreover, the latter is due to the effect of a crowded environment, and not the entanglement of macromolecules as, e.g., in linear polymers. In the case of PAMAM G2, the *G′(ω)* > *G″(ω)* region is also observed (Figure 5c), but it is narrower and shifted to the high-frequency region (2 × 10^11^–4 × 10^12^ rad/s). However, in the case of PPI G2, there is no such region. We believe that this effect is associated with entanglements between dendrimers due to hydrogen bonds, which are quite numerous in PAMAM G2 and practically absent in PPI G2 (see Table 1). The hydrogen bonds were calculated in GROMACS package using standard tools for their analysis. The main parameters for calculating hydrogen bonds were as follows: the hydrogen bond length (i.e., distance between the donor and acceptor) is no more than 0.35 nm, and the hydrogen bond angle (acceptor-donor-hydrogen) is no more than 30 degrees. The distributions of hydrogen bond lengths and angles are shown in Figure 6. It can be seen from this figure that the most probable hydrogen bond length in the PAMAM dendrimer is less than 0.3 nm and the largest number of bonds has an angle of about 15 degrees. These results confirm the presence of hydrogen bonds between the PAMAM dendrimer macromolecules in the melt. However, it is important to note that the simulations were carried out at 600 K, which greatly shortens the average lifetime of hydrogen bonds. According to our estimates, it does not exceed 50 ps.

In this regard, the experimental results obtained for multiarm star polymers with a dendrimer core [63] are of interest, since the hallmarks of both crowded environment and entanglement effects manifest in these systems. Moreover, compared to entanglements, the effect of crowded environment (in this case, jamming) is exhibited in the low-frequency region. This means that our assumptions about the different nature of the *G′(ω)* > *G″(ω)* region for PAMAM G2 and FD G4 are in qualitative agreement with the results of [63]. Thus, the different nature of this region for FD G4 and PAMAM G2 allows us to conclude that these effects manifest themselves in the dynamic characteristics: (i) the crowded environment effect slows down the mechanical relaxation, but at the same time has little impact on the rotational diffusion of the dendrimer; (ii) the entanglement effect slows down both rotational diffusion and mechanical relaxation.

As can be seen from Figure 5, in the high-frequency region, the *G′(ω)* and *G″(ω)* dependences for the studied dendrimers differ from those for PCS. As in the case of the time dependence *G(t)* at short times, these differences are associated with two factors: (i) the PAMAM and PPI dendrimers have less dense cores (with *F_c_* = 2) compared to the PCS dendrimer (with *Fc* = 4) which facilitates the mutual penetration of dendrimer macromolecules (see Figure 4); (ii) the presence of entanglements between the dendrimers due to the formation of hydrogen bonds. The first factor leads to a change in the fluctuation of the dendrimer size: the slope of ⟨δR_g,x_^2^(*t*)⟩ at short times varies from 0.7 (for PCS) to 0.57–0.58 (for PAMAM and PPI). The second factor affects the slope of *G′(ω)* and *G″(ω)* in the high-frequency region. According to the viscoelastic theory [23], an increase in the number of entanglements between dendrimers leads to a slowdown in the growth of both *G′(ω)* and *G″(ω)* in the high-frequency region. Our results are in qualitative agreement with the theory. Since the number of entanglements between dendrimer macromolecules in the PAMAM melt is significantly greater (see Table 1), the slope of *G′(ω)* and *G″(ω)* in the high-frequency region for PAMAM decreases more strongly (up to 0.4) than for PPI (up to 0.45).

## 4. Conclusions

The melts of PPI and PAMAM dendrimers of the second generation (G = 2, with 16 terminal groups) were studied using molecular dynamics simulation. The aim of the work was to study the influence of the chemical structure of the dendrimer on mechanical relaxation. Moreover, for comparison, we used the previously obtained results for PCS G2.

It has been shown that the mechanical relaxation in the tension relaxation region (at high frequencies or short times) of PPI and PAMAM G2 is very different from PCS G2. This is due to the less dense core of PPI and PAMAM than that in PCS, as well as the entanglements between dendrimers due to hydrogen bonds, which are absent in PCS. In this regard, a significant change in the absolute value of the slope of the dynamic modulus from 0.7 (for PCS G2) to 0.4–0.45 (for PPI and PAMAM G2) is observed. However, it is most likely that, for higher generations, the difference in *G(t)* at short times between PCS and the studied dendrimers may significantly decrease or disappear, since the neighboring PPI and PAMAM dendrimer macromolecules will not be able to penetrate into the core region.

In the intermediate and high-frequency regions, the mechanical relaxation of PAMAM slows down compared to PPI and PCS. Moreover, as for PAMAM, the region is observed where *G′(ω)* > *G″(ω)*. This effect is associated with the presence of intermolecular entanglements due to hydrogen bonding. The entanglement between PAMAM dendrimers is also confirmed by the slowdown of rotational diffusion.

Thus, hydrogen bonding strongly affects the mechanical relaxation of dendrimer melts. Therefore, tuning the chemical structure of the dendrimer can be used to control the mechanical properties. We believe that our comprehensive research contributes to the targeted synthesis of dendrimer macromolecules with tailored mechanical properties and the systematization of knowledge about their rheological properties.

## Figures and Tables

**Table 1:** Structural and dynamic characteristics of the second generation PAMAM, PPI and PSC dendrimers. The number of terminal groups, *N_ter_*, the molecular weight, *M*, the radius of gyration, *R_g_*, the rotational relaxation time, *τ_rot_*. The terminal (maximal) time of mechanical relaxation, *τ_max_*, characterizing the tail of the relaxation modulus of dendrimers (*G(t)* ~exp(−2t/*τ_max_*)). The average number of hydrogen bonds per one dendrimer, *<N_HB_*>, within the macromolecule (intra) and number of external hydrogen bonds between macromolecules (inter). Note that the intramolecular hydrogen bond is counted twice (for the donor and acceptor) per dendrimer, as well as the intermolecular hydrogen bond.

Dendrimer	*N_ter_*	*M_d,_* g/mol	*R_g_*, nm	*<N_HB_*>		*τ_rot_*, ns	*τ_max_*, ns	*τ_rot_/R_g_^3^,* ns nm^−3^	*τ_max_*/*τ_rot_*
				intra	inter				
PAMAM	16	3256.2	1.034	10.9	7.5	7.916	4.088	7.16	0.52
PPI	16	1686.8	0.873	2	0	1.074	0.512	1.61	0.46
PCS	16	1964.3	0.872	0	0	0.831	0.461	1.25	0.55

## Data Availability

The data presented in this study are available on request from the corresponding author.
